# A systematic review of objective burn scar measurements

**DOI:** 10.1186/s41038-016-0036-x

**Published:** 2016-04-27

**Authors:** Kwang Chear Lee, Janine Dretzke, Liam Grover, Ann Logan, Naiem Moiemen

**Affiliations:** 1 The Healing Foundation Burn Research Centre, University Hospital Birmingham Foundation Trust, Birmingham, B15 2TH UK; 2 Public Health, Epidemiology and Biostatistics, Institute of Applied Health Research, College of Medical and Dental Sciences, University of Birmingham, Birmingham, B15 2TT UK; 3 School of Chemical Engineering, University of Birmingham, Birmingham, B15 2TT UK; 4 School of Clinical and Experimental Medicine, College of Medical and Dental Sciences, University of Birmingham, Birmingham, B15 2TT UK

**Keywords:** Scar measurement, Burn, Objective measurement, 3D camera, Laser imaging, High-frequency ultrasound image, Colorimeter, Cutometer

## Abstract

**Background:**

Problematic scarring remains a challenging aspect to address in the treatment of burns and can significantly affect the quality of life of the burn survivor. At present, there are few treatments available in the clinic to control adverse scarring, but experimental pharmacological anti-scarring strategies are now beginning to emerge. Their comparative success must be based on objective measurements of scarring, yet currently the clinical assessment of scars is not carried out systematically and is mostly based on subjective review of patients. However, several techniques and devices are being introduced that allow objective analysis of the burn scar. The aim of this article is to evaluate various objective measurement tools currently available and recommend a useful panel that is suitable for use in clinical trials of anti-scarring therapies.

**Methods:**

A systematic literature search was done using the Web of Science, PubMed and Cochrane databases. The identified devices were then classified and grouped according to the parameters they measured.

The tools were then compared and assessed in terms of inter- and intra-rater reproducibility, ease of use and cost.

**Results:**

After duplicates were removed, 5062 articles were obtained in the search. After further screening, 157 articles which utilised objective burn scar measurement systems or tools were obtained. The scar measurement devices can be broadly classified into those measuring colour, metric variables, texture, biomechanical properties and pathophysiological disturbances.

**Conclusions:**

Objective scar measurement tools allow the accurate and reproducible evaluation of scars, which is important for both clinical and scientific use. However, studies to evaluate their relative performance and merits of these tools are scarce, and there remain factors, such as itch and pain, which cannot be measured objectively. On reviewing the available evidence, a panel of devices for objective scar measurement is recommended consisting of the 3D cameras (Eykona/Lifeviz/Vectra H1) for surface area and volume, DSM II colorimeter for colour, Dermascan high-frequency ultrasound for scar thickness and Cutometer for skin elasticity and pliability.

## Background

Burn injury is one of the most common type of traumatic injuries in the world with an estimated incidence of 1.1 per 100,000 population [1] and remains one of the leading causes of deaths, accounting for 5.2 % of 5.1 million deaths due to injuries and violence in 2012 [2]. In the last few decades, major advances in burn care have greatly improved survival rates [3] and an increased number of patients are surviving large burns. Non-fatal burns however is a leading cause of morbidity, as many of these patients develop hypertrophic scars that may lead to significant disfigurement and disability (e.g. contractures).

In order to assess and track the evolution of scars over time, subjective rating scales have been introduced into clinical practice. These scales in general are free or low cost and require minimal training to utilise. Several such scar scales have been developed and are used widely, including the commonly used Vancouver Scar Scale (VSS) and the Patient and Observer Scar Assessment Scale (POSAS) [4]. However, these scar scales are considered to be subjective and the resulting scores can vary between different assessors (inter-assessor variation) [5], different scar severities [6] and age of the scar [7], and some studies have suggested that more than one rater (sometimes as many as five), and utilising the average, is required in order to produce reliable ratings [7, 8]. The POSAS attempts to improve the method of rating scars by including the patients’ perspective; however, patients’ perception and subjective evaluation of their scars have been shown to be influenced by depressive symptoms [9]. The physical characteristics of scars further add to the complexity of rating as changes in both the vascularity and pigmentation can occur simultaneously, and scars are also rarely homogenous in both colour and texture, which makes estimation of mean values difficult and inaccurate for a human observer.

Standardised, quantifiable, reliable (reproducible) and valid assessment tools that provide a more objective evaluation of scars are essential for monitoring the changes in scar quality over time and also to determine the effectiveness of scar treatments.

The various objective measures that relate to scar severity can be divided into the following categories:

- *Colour*: erythema and pigmentation contribute significantly to the appearance of a scar.
- *Dimensions*: it includes planimetry (surface area), thickness and volume.
- *Texture*: surface texture or scar roughness has a significant effect on the patient’s and observer’s opinion of the scar.
- *Biomechanical properties*: it includes pliability and elasticity. Stiffness and hardening of scars are due to increased collagen synthesis and lack of elastin in the dermal layer and can lead to impairment of skin function, especially when the scar is located around joints.
- *Pathophysiological disturbances*: it includes transcutaneous oxygen tension and transepidermal water loss and moisture content.
- *Tissue microstructure*: new non-invasive in vivo imaging techniques analyse the morphological tissue architecture of the scar, providing measurements previously only possible by histopathological analysis of biopsy samples.
- *Pain/sensation*: pain is a commonly measured parameter in many subjective scales however objective methods to measure it are yet to be available. However the measurement of altered sensation may be useful.

In this article, we describe and compare the underlying principles and performance of various currently available objective measurement devices in order to inform clinicians and researchers about their clinical utility for scar assessment. In addition, we discuss innovative technologies that may be applicable to burn scar assessment in the near future.

## Methods

### Criteria for considering articles for inclusion

Published articles that describe non-invasive burn scar measurements were included in this systematic review. Studies that used scar scales which utilise subjective scoring systems were excluded, as studies that made histopathological evaluations of scars via biopsies had no potential to be used in vivo (i.e. requiring the use of ex vivo processing and staining). We chose to include studies comparing the outcomes of wound or scar treatments as well as animal studies and in some cases non-burn scars if appropriate, as excluding these studies may prevent us from identifying new or emerging technologies.

### Search methods

A computerised literature search (until October 2015) was performed using the web-based Web of Science (http://wok.mimas.ac.uk/; years 1900–2015) and PubMed services (www.ncbi.nlm.nih.gov/pubmed/; years 1950–2015) and utilising the Web of Science Core collection and Medline databases. No language limit was set.

The following search strategies were used:

1. (Skin OR derma* OR dermis OR epidermis OR epiderma*) AND (scar OR cicatrix OR fibrosis) AND (objective OR quantitative) AND (burn OR burn$ OR hypertrophic).
2. (Skin OR derma* OR dermis OR epidermis OR epiderma*) AND (scar OR cicatrix OR fibrosis) AND (evaluation OR assessment) AND scale
3. ((burn$ or burn) and hypertrophy)
4. ((burn$ or burn) and (scar or cicatrix))
5. ((scar or cicatrix or fibrosis) and hypertrophy)
6. ((Objective assess* or objective evaluat* or objective measure* or assess$ instrument or assess$ tool or device or measurement system or objective) adj3 assess$)
7. (objective evaluat* or objective measur* or assess$ instrument or assess$ tool or (device or scale or measurement system))
8. NOT (uterus or cardio* or neoplasm or cancer or metastas$ or malignancy)

Web of Science core collection results were further refined by the following terms: surgery or dermatology or critical care medicine or emergency medicine or medicine research experimental or computer science interdisciplinary applications or computer science artificial intelligence or imaging science photographic technology or rehabilitation or medical laboratory technology or engineering biomedical or medicine legal or medical informatics or biophysics or anatomy morphology.

This search produced 5062 articles after duplicates (*n* = 2334) were removed. After filtering by review of titles and abstracts, 151 suitable articles were chosen.

A separate search was also conducted using the PubMed database (www.ncbi.nlm.nih.gov/pubmed) using the following keywords/terms (including MeSH [Medical Subject Headings] terms): skin AND (scar OR cicatrix OR fibrosis) AND (evaluation OR assessment OR assess OR measure OR measurement) AND (objective OR quantitative) AND (burn OR burns OR hypertrophic). A further broader search was conducted using the following keywords and MeSH terms: skin AND (scar OR cicatrix OR fibrosis) AND (evaluation OR assessment) AND scale. No language limit was set. This search retrieved 613 articles, and after filtering by review of titles and abstracts and removal of duplicates, a further 27 articles were included. The reference lists of the selected articles were also searched for suitable studies, and an additional 12 articles were included.

A search of the Cochrane database retrieved no suitable articles.

A grey literature search was performed using the Bielefeld Academic Search Engine (BASE) database with the term “objective measurement of scarring”. This search included books, reports, papers, lectures, theses, reviews, and primary data document types and excluded article, journals, audio, videos, images, maps, software and sheet music document types. This search produced 180 hits (after 50 duplicates removed), and after review, 6 articles were deemed suitable for inclusion into the review.

Full text articles were obtained for the articles where possible, and a further 28 records were removed after evaluating the full text. Articles which were only available in abstract form and had no extractable data were also excluded.

Thus, the total number of articles selected for review was 157. This includes 9 review articles.

**Fig. 1:**
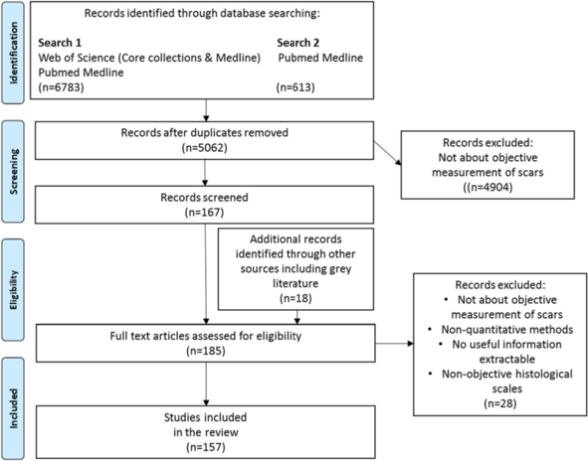
Preferred Reporting Items for Systematic Reviews and Meta-Analyses flowchart

The selection process for the eligible articles is outlined in Fig. 1 below.

### Quality assessment

The validity and reproducibility of the devices were evaluated when statistical data were available especially in terms of reproducibility of the assessments. Where available, the additional value of the device compared with subjective scar scales and/or other tools is discussed.

In terms of interpreting the intra-class correlation coefficients (ICC), some guidelines have been provided by Landis and Koch [10] for Kappa coefficients (which are also reasonable for the ICC) suggesting that:

- Kappa of <0.00 indicates “poor” agreement
- Kappas from 0.00 to 0.20 indicate “slight” agreement
- Kappas from 0.21 to 0.40 indicate “fair” agreement
- Kappas from 0.41 to 0.60 indicate “moderate” agreement
- Kappas from 0.61 to 0.80 indicate “substantial” agreement
- Kappas from 0.81 to 1.00 indicate “almost prefect” agreement

However, it should be noted that these guidelines are subjective.

Feasibility of devices was assessed via the commercial availability, portability and cost of the devices.

An economical assessment of the devices based on the literature was not possible due to the lack of such data in the articles; however, several of the companies with commercially available devices were contacted to provide quotes, and although it was not possible to publish the exact prices due to confidentiality issues, the devices are categorised into price ranges (<£5000, £5000–10,000, >£10,000, >£30,000).

## Results

Articles, reviews and editorials that described objective burn scar assessments were retained. These were then classified into six categories based on the assessed variables: (1) colour, (2) scar dimensions (e.g. thickness or height, surface area), (3) texture, (4) biomechanical properties (e.g. elasticity, pliability), (5) physiological disturbances (e.g. hydration) and (6) non-invasive morphological imaging techniques.

### Colour

Colour is a major factor that affects the aesthetics of a scar and is mainly composed of two components: melanin (the brown pigment made by activated cutaneous melanocytes) and erythema (the redness that is caused by haemoglobin in the dilated/congested remodelled cutaneous vasculature). Other pigments that localise in scars, such as bile and carotene, may also contribute to the overall appearance of the scar. Colour measurements can be used to gauge the effectiveness of anti-scarring treatments since they reflect abnormal skin architecture/composition [11]. Measurement of the scar colour can be complicated by several factors, such as skin layer thickness, reflection from the skin surface and environmental factors including light and temperature. The measurement of erythema is further influenced by patient-related factors such as activity and positioning of affected areas as such movements may affect the blood circulation and hence the erythema of the skin.

Although visual assessment of colour has been incorporated into various scar scales, it is a subjective evaluation method that provides relative rating systems. Even in normal circumstances, the human brain cannot accurately quantify colour or its intensity. A famous recent example of this is the “blue and black dress” which shows that human colour discrimination may be affected by the illuminant colours, level of ambient illumination and the background colours of a visual display terminal [12, 13]. Neuropsychiatric conditions have also been shown to affect colour discrimination [14]. In scars, changes in vascularity and pigmentation occur simultaneously and overlap each other which make colour observation and reporting even more difficult for a human observer, e.g. it is difficult to assess the pigmentation of a scar in a highly vascularised scar as the erythema would obscure the increase or lack of pigment. Additionally, as scars often have an uneven colour distribution, human observers cannot easily or accurately provide a mean value for a certain area.

More recently, several objective and reproducible methods of colour evaluation have been developed and they can be broadly classified as follows:

- *Reflectance spectroscopy*: tristimulus reflectance colorimetry and narrow-band spectrophotometry
- *Laser imaging*: it measures the microcirculation in the scar which influences the erythema of the scar.
- *Computerised analysis of digital photographs*: it can include two-dimensional (2D) and three-dimensional (3D) images which are then digitally analysed to quantify colour values.

#### Reflectance spectroscopy

**Fig. 2:**
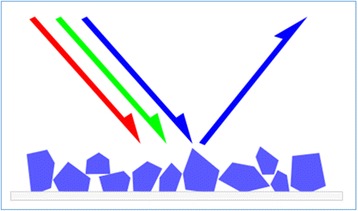
Graphical illustration of the concept of reflectance spectroscopy. (Source: http://commons.wikimedia.org/wiki/File:Simple_reflectance.svg)

Reflectance spectroscopy is a well-established technique of more than 50 years [7] and currently one of the most commonly used methods for measuring colour. Techniques that utilise reflectance spectroscopy quantitatively measure the colour and intensity of reflected light. For example in Fig. 2, when light consisting of red, blue and green is shone upon a surface, if the material absorbs red and green light, then only the blue light is reflected which will make us perceive the material as blue. A biological example is the detection of the oxygenation of haemoglobin. When haemoglobin is illuminated with white light, oxygenated haemoglobin will absorb a higher proportion of blue light and reflect back red light whereas de-oxygenated haemoglobin absorbs more red light and thus appears bluer. In reality, the process is more complicated as the light that is shone (termed incident light) onto biological tissues can be reflected in many different trajectories, and this scattering also influences our perception of the colour of an object.

Tristimulus reflectance colorimetry and narrow-band simple reflectance (or spectrophotometry) are both based on the principle of reflectance spectroscopy.

**Fig. 3:**
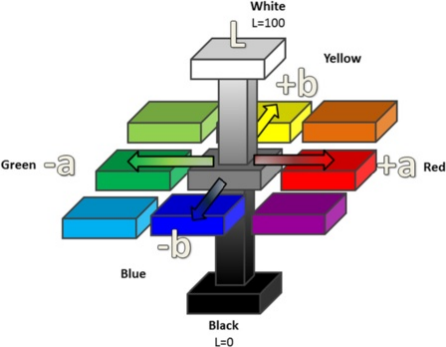
Graphical representation of the L*a*b* colour measurement system. (Source: Kwang Chear Lee)

Tristimulus reflectance colorimetry [15] describes colour by three values: L* (clarity, lightness or brightness); a*, the amount of red or green (erythema); and b*, the amount of yellow or blue (pigmentation) (see Fig. 3). For example, a white coloured object would have a higher L* value compared to a darker coloured object and a scar that it is redder than normal skin would give a higher a* value than normal skin. Additionally, another approach to quantify colour is by using the saturation or chroma of colour (C∗) which is a vector magnitude in the chromatic plane calculated from a* and b* values [16, 17].

There are currently several spectrocolorimeter devices that utilise the principle of tristimulus reflectance colorimetry, including the Minolta Chromameter [15, 18, 19] (Minolta Camera Co., Osaka, Japan), the Labscan XE [17] (Hunter Associates Laboratory, Inc., Reston, VA), DSM II Colormeter [20], NF-333 [21] (Nippon Denshoku Co. Ltd, Japan), Micro Color (Dr. Bruno Lange GmBH, Dusseldorf, Germany) [22], X-Rite SP64 Spectrophotometer (X-rite Inc, Michigan, USA) [23] and the Visi-Chroma VC-100 (Biophotonics, Belgium) [22, 24]. Camera systems such as the Eykona 3D camera can also be calibrated to report colour values using the L*a*b* system [25]. However, a drawback of the Eykona 3D camera is that although its cost is low, it currently requires consumables in the form of one-use targets (about £70 for 25 targets) that have to be placed next to the area of interest when taking an image, although there are plans to introduce reusable targets in the coming months according to the company.

A study by Li-Tsang et al. [17] showed that the intra- and inter-rater reliability for the Labscan XE device for hypertrophic scars was satisfactory, with an intra-class correlation coefficient (ICC) ranging from 0.95 to 0.99 for intra-rater reliability, and 0.50 to 0.99 for inter-rater reliability in all the three colour parameters (L*, a* and b*). A strong positive correlation was also found between VSS scores and the readings obtained from the Labscan XE device. The device that was utilised in the literature was not portable; however, newer portable versions are currently available. A study by Draaijers et al. showed that the overall evaluations of scar colour with both the Dermaspectrometer and the Minolta Chromameter are more reliable than the visual evaluation and scoring of scar colour carried out by observers using a 10-step score, whereby a score of 1 reflects normal skin and a score of 10 reflects the worst scar imaginable [15]. However, devices that rely solely on tristimulus colorimetery have been shown to have poor correlation scores with patient scar scales when measuring pigmented or hypo-pigmented scars due to the scar scales scoring hyper- and hypo-pigmented scars higher as deviations from normal skin.

Narrow-band spectrophotometry [15] devices on the other hand measures the vascularisation and pigmentation of the scar based on differences in red and green light absorption by haemoglobin and melanin, respectively. The Dermatospectrometer (or the newer version, DSM II Colormeter) [20, 26, 27] (Cortex Technology, Hadsund, Denmark) and Mexameter [20, 28] (Courage + Khazaka, Germany) are examples of a device that uses this principle. In comparison with the Minolta Chromameter and Labscan XE, the Dermaspectrometer is a smaller and hence a more portable device and the use of the erythema and melanin indexes is less complicated to understand and analyse compared to the L*, a* and b* of the Minolta Chromameter. It has also been shown to have a slightly better correlation with clinical scores when compared with the Chromameter [29]. Unfortunately, the Dermatospectrometer has been withdrawn from the market but it has been replaced with a newer model, the DSM II Colormeter. The DSM II Colormeter [20] (Cortex Technology, Denmark) is a small, fully hand-held device that utilises both tristimulus colorimetry and narrow-band spectrophotometry technology and produces reliable readings [20]. It has an improved utility, in terms of cost and assessment time as it utilises one instrument instead of two to obtain both tristimulus colorimetry readings as well as narrow-band spectroscopy readings. The Mexameter also has good intra-observer and inter-observer reliability in scar assessments [20].

Caution however must be used when using erythema to grade the severity of scars. This is because scars can often be very vascular initially but this does not mean that they will become hypertrophic, e.g. in the study by Nedelec et al. [30], the Mexameter was unable to differentiate hypertrophic scars from normal scars as donor sites were very erythematous, but we know that donor sites rarely progress to become hypertrophic scars.

A common disadvantage of all of the aforementioned devices is that they employ a small measuring area, e.g. the measuring area for the Minolta Chromameter is only 3 mm [18] and the other devices range from 5 to 8 mm [7]. Therefore, multiple measurements, especially in larger scars, need to be performed to provide accurate scores, but these increase the risk of observational bias. Additionally, these devices also require contact with the skin which can change the colour if too much pressure is applied. Environmental lighting may also affect the readings obtained, although many of the companies of these devices (e.g. DSM II Colormeter) claim that the flash that is utilised by these devices is strong enough to overcome and compensate for any differences in colour caused by indoor lighting.

#### Large area spectrophotometry

Some investigators have attempted to overcome the problem of small measurement areas by utilising camera systems to allow the imaging of larger areas. Cheon et al. utilised digital photographs taken with a digital camera (Nikon D70s, Tokyo, Japan) under the same light source and obtained L*a*b* values for the regions of interest (whole scar lesions when possible) using Adobe Photoshop (Adobe systems Incorporated, San Jose, CA). The test–retest consistency (or intra-rater reliability) of the L*a*b* as determined by the intra-class coefficient ranged from 0.95 to 0.99 and the inter-rater reliability was also good with values ranging from 0.94 to 0.98 [31, 32].

Another method of spectral modelling developed by Kaartinen et al. utilises standardised digital imaging (SDI) with computer controlled lighting to quantify colour changes [7, 8, 33]. This system allows a larger area of the skin to be analysed with an only slightly weaker accuracy compared to the previously mentioned spectroscopy-based systems [33]. This method, however, is yet to become commercially available, but a similar system, Scanoskin (Leniomed Ltd, London, UK), is available. The Scanoskin system utilises polarised light, which has the advantage of blocking the reflectance from the skin which allows better analysis of the epidermal and superficial dermal layers [34]. The system is currently used only to assess burn depth via the imaging of haemoglobin (erythema/vascularisation) and haemosiderin or melanin. Images which are taken (with a modified SLR camera with polarised lenses) are processed by the provided software which splits them into separate erythema and melanin components. Quantification of erythema and pigmentation (melanin) has to be performed on the exported images using software such as ImageJ [35–37].

The evidence for using objective measures in measuring colour is encouraging and is based on a relatively small number of studies, and more research is needed [38].

#### Spectrophotometric intra-cutaneous analysis (SIA)

Analysis of colour information purely in the visible spectrum is insufficient to provide information relating to a lesion’s deeper structures, and it was this realisation that prompted research at the University of Birmingham to extend the spectrum of light used into the infrared region (700–1000 nm). Spectrophotometric intra-cutaneous analysis via the clinical device, SIAscope, utilises a probe (12 × 12 mm or 24 × 24 mm) that utilises radiation ranging from 400–1000 nm and produces 8 narrow-band spectrally filtered images of the skin which are then processed by software algorithms and allows the visualisation and quantification of melanin, collagen and blood [39]. Although developed for diagnosing skin cancers, it can and has been used to monitor the changes in scar tissue in response to treatment [40].

#### Computerised analysis of digital photographs

Digital photographs can be taken with any standard digital camera, e.g. the Nikon 8400 [19].

Photos are then downloaded for analysis by proprietary software packages such as KS400 (Kontron Electronic GMB, Carl Zeiss Micro-Imaging, Inc., Thornwood, NY, USA) [41] or the freely available ImageJ. One study utilised an artificial neural network to perform chromatic analysis of the digital image of a burn scar [42]. Colour measurements using ImageJ have been shown to be equivalent to those obtained using a colorimeter (Chromameter, Konica-Minolta) [19]. Several studies have attempted to improve the objectivity of photograph analysis of scars by standardising factors such as distance and lighting [19] or using computerised image capturing systems [43–45]. However, even this method fails to allow scars to be compared objectively as humans vary in terms of how we set the measurement criteria for and analyse colour [29, 46] and the photographs have been shown to have limited utility when assessed using computer-based subjective scales [47]. Improved computer programmes may overcome the limitations of the human brain and provide objective analysis of the digital photographs. However, computer programmes cannot properly “see” colour and thus have to convert colour information into digital data, thereby losing valuable information.

Computer programmes utilise two methods to analyse colour. The hue-saturation-value (HSV) method analyses colour by separating it into three main components: hue (dominant wavelength), saturation (amount of white) and value (amount of black). The other method utilises colour models of which there are two main ones: the Red, Green and Blue (RGB) model and the Cyan, Magenta, Yellow and Black (CMYK) model. Measurement techniques using other systems such as the L*a*b* system have also been described [48].

To remove the influence of light and camera settings, generally a card carrying standard colours (e.g. Pantone colour chart [Pantone Inc, USA] [16], Macbeth Digital Colorchecker SG colour chart [Munsell Colour services Laboratory, X-Rite Inc, Michigan, USA] [25, 44]) is recommended to be placed beside the scar being photographed so that every photo taken would include areas of known colour properties, allowing an objective colour evaluation [16].

Table 1 summarises the colour measurement devices in terms of parameter measured, reliability, correlation with clinical score and cost.

**Table 1:** Comparison of colour measurement devices in terms of parameter measured, reliability, correlation with clinical score and cost

Device	Company	Parameter	Intra-rater Reliability	Inter-rater reliability	Correlation with clinical score	Cost	Portability	References
Computerised colour analysis	Sony Hi-8 Handycam CCD-TR 705E video camera recorder and Adobe Photoshop	Hue, saturation, value	No data	No data	VSS Vascularity score significantly correlated with hue (*r* = 0.311) and saturation (*r* = 0.35) (*p* < 0.051), index with hue and saturation combined correlated even better (r = 0.42).	£5000–10,000	Yes	Davey et al. 1999 [16].
Computerised colour analysis	Nikon D70 camera and Adobe photoshop	Tristimulus colorimetry (L*a*b*)	0.95–0.99	0.94–0.95	L* and a* values are more important than b* values in distinguishing colour features between normal skin and scars.	No data	Yes	Cheon et al. 2010 [32]
Labscan XE (non-portable version)	Hunterlab	Tristimulus colorimetry (L*a*b*), chroma and hue.	Good (0.95–0.99)	Acceptable to good (0.50–0.99, outlier low value of 0.50 for a* [ranged from 0.01 to 0.77])	L*, a*, b* and hue had moderate to strong correlation with VSS pigmentation and vascularity scores. Chroma had low correlation with pigmentation and vascularity (*r* = −0.40 and −0.17)	>£10,000	Poor	Li-Tsang et al. 2003 [17]
Labscan XE (portable version)	Hunterlab	Tristimulus colorimetry (L*a*b*)	No data	No data	No data	£5000–10,000	Yes	Li-Tsang et al. 2005 [102]
Chromameter	Konica-Minolta	Tristimulus colorimetry (L*a*b*)	Acceptable (0.73–0.89)	Good (0.91–0.97)	Unable to differentiate between hypo- and hyperpigmented scars and normal and 'red' skin (On Seattle, Hamilton and Vancouver scar scales)	£5000–10,000	Yes	Draaijers et al. 2004 [15], Oliveira et al. 2005 [29],
Eykona 3D camera	Fuel 3D	Tristimulus colorimetry (L*a*b*)	No data	No data	Good correlation (Manchester scar scale)	<£5000 (not including targets)	Yes	Hallam et al 2013 [25]
Colorimeter	Courage + Khazaka	Tristimulus colorimetry (L*a*b*) and ITA (Individual Typology Angle)	No data	Good (0.91–98)	No data	£5000–10,000 (including cost of hub)	Yes	van der Wal et al. 2013 [20].
Mexameter	Courage + Khazaka	Narrow-band spectrophotometry (melanin and erythema)	Good for melanin (0.89–0.97) and acceptable for erythema (0.74–0.90)	Good for melanin (0.95) and erythema (0.82–0.85)	No data	<£5000	Yes	Nedelec et al. 2008 (I) [30], Nedelec et al. 2008 (II) [106], van der Wal et al. 2013 [20].
Dermaspectrometer/DSM II Colorimeter	Cortex	Both tristimulus colorimetry and narrow-band spectrophotometry	Erythema: 0.29–0.94 Melanin: 0.72–0.87 L*a*b*: no data.	Erythema: 0.68–0.91 Melanin: 0.91–0.94 L*a*b*: no data.	Erythema: Moderate but significant *r* = 0.50 (<0.001) Melanin: weak but significant *r* = 0.32 (0.02)–0.63 (<0.001)	<£5000	Yes	Gankande et al. 2014 [6], Gankande et al. 2015 [248], van der Wal et al. 2013 [20], Oliveira et al. 2005 [29].
Standardised digital imaging (SDI) + Spectral modelling (SpM)	Custom made	Estimated concentration change of haemoglobin and melanin	Good for haemoglobin (0.875) and melanin (0.886)	Good for haemoglobin (0.955) and melanin (0.959)	Acceptable correlation with POSAS (0.63 for haemoglobin, 0.60 for melanin) and VSS (0.74 for haemoglobin, 0.53 for melanin)	<£5000 (but not commercially available)	Yes	Kaartinen et al. 2011 [7, 8].
Dermoscopy	Hong Kong Productivity Council	RGB values: lightness and redness	Redness: 0.980	Redness: 0.93	Strong correlation between the VSS scores of vascularity and the RGB values of redness obtained from the dermoscope (*r* = 0.625, *p* < 0.01). Strong correlation also found between transformed VSS scores of pigmentation and the lightness of the dermoscope pictures when vascularity was blanched out (i.e. when measuring pure pigmentation) (*r* = 0.783, *p* < 0.01).	No data	Yes	Wei et al. 2015 [238].
			Lightness: 0.965	Lightness: 0.871				

*RGB *= red, green and blue

#### Laser imaging

The amount of haemoglobin or erythema present in a scar can be measured indirectly via laser imaging [49, 50] that measures the blood flow in a scar. Immature scars show a significantly increased blood flow due to their higher vascularity compared to mature scars. Increased microcirculatory blood flow (as measured by Laser Doppler Flowmetry (LDF)) has also been shown to be a potential indicator for the occurrence of hypertrophic scarring [51]. Hypertrophic scars will typically generate readings that are two to three times greater than that made in normal skin [50, 52, 53] and four times greater than that in a non-hypertrophic scar [50]. Laser-based methods have the advantage of being fast, reproducible and having a good correlation with the VSS; however, they are subject to structural changes in the skin and environmental and body temperature fluctuations [54–56].

Laser-based methods can be divided into three techniques: LDF, Laser Doppler Imaging (LDI) and Laser Speckle Imaging (LSI)/Laser Speckle Perfusion Imaging (LSPI). With the older Laser Doppler Flowmeter, the fibre optic probe is in contact with the tissue surface and is a single-point measure [49, 57]. Laser Doppler Flowmeter [29, 52, 55, 56] systems, such as the DRT4 [53] (Moor instruments, Devon, UK) or the LaserfloBPM [58] (Vasamedics Corp, St Paul, Minnesota, USA), the fibre optic probe is in contact with the tissue surface and provides a single-point measure of an indirect evaluation of scar colour by measuring the cutaneous bloodflow present in a scar [49, 57]. LDF systems are more limited compared to the other laser-based methods (see below) as they measure flow within a small area and, thus, are unsuitable for use with larger, heterogeneous scars.

In contrast, Laser Doppler Imaging (LDI) devices, such as the Lisca PIM1.0 imager (Lisca Development AB, Linköpen, Sweden) and The Moor LDI (Moor Instruments, Devon, UK) [49], utilise a laser beam to scan several points across a tissue surface and generates a 2D colour-coded image that is correlated to the blood flow [49]. They are primarily used for burn depth assessment but have been utilised for scar evaluation [49, 59]. The method is, however, hampered by long measurement times and low resolution [57]. LSI and LSPI are alternative perfusion monitoring techniques that generate rapid, high-resolution images of tissue. As red blood cells move during circulation, dynamic interference patterns that change with time are created. Blood flow maps can then be created from the coherent light that is reflected from stationary tissue, generating a high contrasted speckle pattern that remains static in time. As indicated previously, high measurements reflect high blood flow and immature/hypertrophic scars. LSI devices compare favourably with the more established LDI instruments, but offer advantages in terms of a faster scan time, higher resolutions and the ability to zoom in with increased resolution of a smaller field of view, a feature that is not possible with LDI [57, 60].

A major disadvantage common to all laser imaging systems are that they are not very portable (with the exception of a new commercially available laser speckle imaging device developed by Moor instruments [61]) due to their size and are often very expensive, with costs of >£30,000.

Table 2 summarises the comparison of laser devices in terms of parameter measured, reliability, correlation with clinical score and cost.

**Table 2:** Comparison of laser devices in terms of parameter measured, reliability, correlation with clinical score and cost

Device	Company	Parameter	Intra-rater reliability	Inter-rater reliability	Correlation with clinical score	Cost	Portability	References
Laser Doppler Flowmeter	Moor	Blood flow	No data	No data	LDF showed significant difference in blood flow within hypertrophic and keloid scars and normal skin (2.6–2.8-fold higher).	>£30,000	Poor	Clark et al. 1996 [56], Timar-Banu et al. 2001 [53].
Laser Doppler Imaging	Lisca, Moor	Blood flow (red and near infrared wavelengths)	No data	No data	Correlations with clinically assessed grades (VSS) of pigment, vascularity, pliability, and height ranged from *r* ^2^ = 0.63 to 0.95.	>£30,000	Poor	Stewart et al. 2005 [60] Bray et al. 2003 [49].
Laser Speckle Perfusion Imaging	Moor	Blood flow	No data	No data	Correlations with clinically assessed grades (VSS) of pigment, vascularity, pliability, and height ranged from *r* ^2^ = 0.73 to 0.94.	>£30,000	Poor	Stewart et al. 2005 [60]

*LDF* = Laser Doppler Flowmetry; *VSS* = Vancouver Scar Scale

#### Thermographic analysis of burn scars

Thermographic cameras detect radiation in the long-infrared range of the electromagnetic spectrum (9–14 μm) and can be used to produce images or videos of that radiation. Thermography can be divided into passive (where the object can be imaged directly as it has a higher or lower temperature than the background) and active thermography (where an energy source is required to produce a thermal contrast between the imaged object and the background). Several studies have looked at using thermography to assess the depth of burn wounds [37, 62–64].

Our literature search however has only been able to identify one small study done in 1985 (*n* = 12) which utilised thermographic analysis of the scar temperature in an attempt to differentiate hypertrophic and non-hypertrophic scars [65]. No relationship between scar temperature and hypertrophic scar formation was found.

A more recent case report by Horta et al. [66] which utilised a thermography camera (FLIR SC7000 thermography camera; FLIR Systems, Wilsonville, OR, USA) showed that factors such as muscle activity or the lack of mucosa, cartilage and bone can influence the thermographic reading of scars rather than the degree of hypertrophy itself. This further complicates the use of thermography to objectively quantify scars.

### Scar dimensions

#### Surface area and volume

Planimetry is the measure of the surface area of a scar and, when done over time, can be used to assess the contraction or expansion of a scar.

The most basic method of planimetry, that does not require specialist equipment or trained personnel, is the linear method where the maximum length and width of the wound is measured directly on the patient and the surface area is then calculated by multiplying the maximum length and width. As can be expected, this technique is inaccurate as scars are rarely rectangular or square in shape and will produce results that are significantly different from those obtained with tracing and photography methods [67].

The second method involves the tracing of scar margins either on sheets of paper, clear plastic film or any transparent non-stretchable material [27, 46]. The surface area traced on these sheets can then be calculated by outlining wound margin with the tip of a planimeter (Koizuni Sokk Manufacturing Ltd., Nagoaka-shi, Japan) [67] or by digitising the tracings on these sheets and using software such as NIS-Elements (Nikon, Amstelveen, The Netherlands) [27] , ImageJ [68] or Digimizer software [69] to calculate the surface area. Dedicated systems have also been developed such as the Visitrak (Smith & Nephew) which have been shown to have high intra- and inter-rater reliability and high validity in the measurement of the surface area of ulcers [70] although the maximum size of the area that can be measured at a time is limited by the disposable tracing grid used (14 cm × 14 cm).

**Fig. 4:**
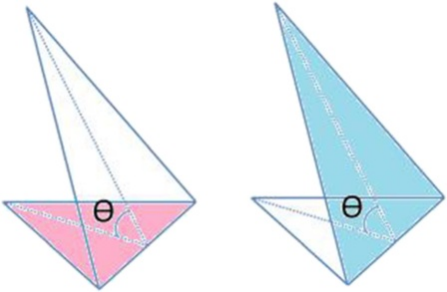
The 2D or planimetric area (in *pink*) is always smaller than the 3D area (in *blue*). (Source: Kwang Chear Lee)

The third method uses digital photography combined with image analysing programmes such as ImageJ, Image Tool (C.D. Wilcox and colleagues, San Antonio, TX, USA) [29] or Adobe Photoshop (Adobe Systems Inc., San Jose, California, USA) [71] to measure the surface area. A significant problem with 2D photography is that it is subject to parallax errors and projecting a three-dimensional object onto a two-dimensional image. Due to this, the 2D surface area (or planimetric area) calculated does not take into account the wound surface topography and will nearly always underestimate the true three-dimensional surface area (see Fig. 4).

With smaller scars, this error would be small but will increase as the size increases. A study by van Zuijlen et al. compared the direct and indirect (through 2D photography) tracing methods [71]. It found that both techniques were reliable (*r* ≥ 0.82, *p* < .001) for surface lesions with a scar surface area of 25 cm^2^, but planimetry by photography was superior to planimetry by direct tracing in respect to inter-observer reliability for surface lesions of 50 and 75 cm^2^, with increasing scar size resulting in decreasing inter-observer reliability. However, planimetry by direct tracing was more accurate on curved surfaces (e.g. forearm), with a statistically significant reduction of the surface area obtained when compared to results with planimetry after photography. The use of photography [29, 43] to measure surface area, although useful, is subject to variance caused by lighting conditions, distance and camera settings and does not provide any information on volume.

Three-dimensional (3D) measurement systems can overcome the limitation of 2D photograph, and in addition to surface area measurements, the 3D camera systems are also able to measure the volume of scars much more quickly and easily compared to traditional moulage and moulding [72] methods.

A 3D image can be achieved via various methods. A method that is commonly used in the medical is stereo-photogrammetry. These systems are non-contact and involve taking two or more pictures using either one or multiple cameras which can be on the same device (e.g. Eykona wound measurement system, Fuel 3D, UK [25]) or separate devices (e.g. 3D MD static systems, 3dMD, USA [73]). Some authors have even developed their own systems with standard cameras (e.g. Stereoimage optical topometer (Korea University, Seoul, South Korea) with PC vision plus (AES, Sydney, Australia)) [74]. Other devices utilise mirrors to achieve a similar effect for, e.g. LifeViz I, II, Mini or Micro (Quantificare S.A., Sophia Antipolis, France) [75, 76] and Vectra H1 3D imaging system (Canfield Scientific Inc, Fairfield, NJ, USA) [77–80]. Other systems utilise the projection of a complex speckled pattern in combination with a colour camera to produce the 3D images [81].

These software are able to provide information about the surface area [82] and tissue volume above the skin [83] (including correction for curved surfaces) as well as geometry, texture and, as mentioned above, the colour of scars for which the performance of the Eykona device has been shown to compare favourably with the subjective Manchester Scar Scale (MSS) [25]. These devices share a few common drawbacks. Firstly, none of them have been validated in scar studies, but their ability to measure the area [83–85] and volume of wounds and tissue (e.g. breasts [82]) has been shown in other non-scar related studies. Additionally, the maximum area that can be imaged is limited to the size of about an A4 size sheet of paper which is not ideal for large burns scars. Furthermore, although stitching of images is possible, this really only applies to the face as it is easy to identify anchor points such as the eyes and nose, but to do so for other highly curved surfaces such as the forearm or the whole body would be technically challenging and time consuming and requires high-end hardware and thus a true 360° view would not be easily possible [80]. Hairy areas of the body can also pose a problem [80].

The Lifeviz and Vectra H1 systems have an advantage over the Eykona in that they have adjustable light-beam pointers to aid positioning and do not require one-use disposable targets which the Eykona system does but they are also significantly more expensive. Furthermore, the Eykona is no longer being developed by the company and has not been updated recently, thus its resolution is significantly lower (250 micron sampling via two 5 MP sensors) [86] compared to the Lifeviz Mini (13.5–24 MP, 0.5–2 mm geometry resolution) [87] or Vectra H1 cameras (18 MP, 0.8 mm geometry resolution) [88].

More recently, light field or plenoptic technology has been introduced. Cameras utilising this technology (Raytrix 3D camera systems, Raytrix, Germany [89]) capture information about the intensity and also direction of the light rays utilising an array of micro-lenses [89]. The images or data are then processed and merged using dedicated image analysis software into a single 3D image. Additionally, other commonly used 3D imaging techniques include structured light scanner systems (or coherence scanning interferometery) such as the Artec [90] (Artec Group,USA/Luxemborg/Russia) and ATOS series of scanners [91], and laser scanning devices such as the Minolta Vivid 900 or 910 3D linear laser scanner (Konica-Minolta, Osaka, Japan) [92, 93]. Whole body scanners such as the Cyberware Whole Body Color 3D Scanner (Model WBX, Cyberware Inc, Monterey, California) [94] are also available. These other systems have the ability to scan much larger areas (up to the size of a car with some systems) compared to the Eykona, Lifeviz and Vectra; however, they have not been specifically manufactured or optimised for medical use. For example with the Artec Eva system, the software supplied is able to calculate the surface area and volume of an object on a flat surface but not on curved surfaces. Specialised 3D analysis software such as Rapidform (Inus technology, Seoul, South Korea) [93] is required to measure and quantify surface area and volume information obtained from these scans. The authors are not aware of any published studies that have validated the surface area and volume measurements produced by these devices or software.

A different approach to calculating the surface area of scars is through the use of a combination of 2D photography and 3D models. The Burncase 3D (RISC Software GmbH, Austria) software has been developed for the estimation of burn surface areas primarily but it theoretically can be adapted to measure the surface area of scars. With the Burncase 3D programme, 2D photographs of the lesions are superimposed onto a 3D model that can be adjusted according to the height, weight, age and gender of the patient. The outline of the lesion is then traced onto the 3D model from the photographs (which can be multiple and is aided by an automated alignment algorithm that uses corresponding landmarks to allow quick matching [95]) and the software then estimates the surface area. The areas can also be classified into different categories if needed (e.g. normal and hypertrophic scar areas) and thus useful to track the progression of the wounds from time of burn through to scar formation. As it uses standardised 3D models to estimate surface area, much work is still required to validate the accuracy and precision especially in small children (currently in progress [96, 97]) and obese patients [95]. In a study which utilised mannequins, the inter-class correlation between the single raters of the mean percentage of artificially created burn areas was 0.988 with relative underestimations of burn wound areas of 0.4 % in the child mannequin, and overestimations of 2.8 and 1.5 % for the female and male mannequins when compared to areas as measured with 2D planimetry imaging [97]. 

Table 3 below summarises the comparison of 3D measurement devices in terms of parameter measured, reliability, correlation with clinical score and cost.

**Table 3:** Comparison of 3D measurement devices in terms of parameter measured, reliability, correlation with clinical score and cost

Device	Company	Parameter	Intra-rater Reliability	Inter-rater reliability	Correlation with clinical score	Cost	Portability	References
Eykona 3D camera	Fuel 3D	Surface area and volume	Intra-operator variability: area: 0.9 %; volume: 4.0 %	Intra-operator variability: area: 1.7 %; volume: 4.0 %	No data	<£5000 for the camera unit.	Yes	Paterson et al. (Eykona Medical Imaging FAQ) [86].
						~£3 for each disposable target but device can now be configured to use reusable targets.		
Lifeviz I, II, Micro	Quantificare	Surface area and volume	No data	Surface area: ICC = 0.99 (Coefficient of variation 5.9–6.8 %)	Surface area: Excellent level of agreement with Visitrak (ICC 0.96, 95 % CI 0.93, 0.97); however greater level of variability in larger wounds especially circumferential wounds. Volume: *r* ^2^ = 0.9678 when correlated with actual volumes of model scars	£10,000–£15,000	Yes	Lumenta et al. 2011 [76], Stekelenburg et al. 2013 [75].
				Volume: no data				
Vectra H1	Canfield Imaging Systems Inc.	Surface area and volume	No data	No data	No data	£10,000–£15,000	Yes	Tzou et al. 2014 [256], Urbanova et al. 2015 [80].
Artec Eva	Artec	Surface area and volume	No data	No data	No data	<£10,000 (depends on package)	Yes	N/a
Minolta Vivid 910 3D linear laser scanner	Konica-Minolta	Surface area and volume	No data	No data	No data	>£15,000	Yes	Taylor et al. 2007 [93].
Moulding (positive–negative moulage)	N/a	Volume	ICC = 0.921–0.995	ICC = 0.759–0.977	No data	Dependent on moulding material and measurement techniques used	Yes	Berman et al. 2015 [72].

#### Thickness

The accuracy of subjective estimation of scar thickness has been shown to be quite low, 67 % (when measured against ultrasound measured thickness) [98] and thus unreliable.

Objective thickness or height of a scar can be evaluated by measurement by 3D photography (see above) or the use of negative–positive moulage [99]. A negative–positive moulage is performed by firstly making a negative impression cast (negative moulage) of the scar using materials such as alginate, silicon, siloxane [58, 72], dental impression material [100] or plaster of paris. A positive impression cast (positive moulage) is then made by pouring a material that will harden (e.g. plaster of paris, wax) into the negative moulage. Once hardened, this positive moulage can then be measured. These techniques have some limitations and are inaccurate as the portion of the scar below the surface of the skin is not included in the measurement [101].

This limitation can be overcomed by using high-frequency (5–20 MHz) ultrasound systems such as the Tissue Ultrasound Palpation System (TUPS; Biomedical Ultrasonic Solutions, Hong Kong) [102–105] , the Dermascan C [30, 53, 106, 107] (Cortex, Hadsund, Denmark) devices, Acuson Sequoia 512 [108] (Siemens, Germany; highest frequency probe available is 10 MHz), HDI 5000 (Philips, Amsterdam, Netherlands) [109], and the Dermcup 2020 (Atys Medica, Soucieu-en-Jarret, France) [110]. High-frequency ultrasound systems have previously been used in many dermatological applications [111].

**Fig. 5:**
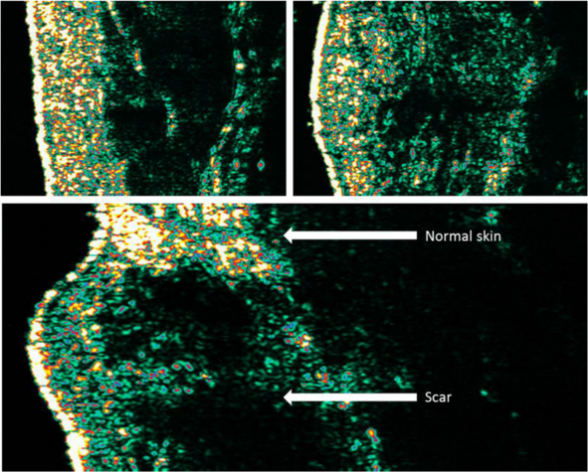
High-frequency ultrasound image of normal skin (*top left*, site: forearm). High-frequency ultrasound image of hypertrophic scar (*top right*, site: shoulder). High-frequency ultrasound image of normal skin (*top*) and adjacent scar tissue (*bottom*) (*bottom*, site: shoulder). Note that the scars appear more hypo-echoic as it is more homogenic and thus appears darker. Colours represent the intensity of the acoustic signal with bright colours (*yellow*) representing high-intensity and darker colours (e.g. *green*, *black*) representing low-intensity areas. (Source: Kwang Chear Lee, taken with Dermascan C)

Ultrasound skin imaging is performed by firing an acoustic pulse into the skin and measuring the acoustic response from the skin which is picked up by an ultrasound transducer. The signals are then processed, and a cross-sectional image is produced which represents an intensity/amplitude analysis of these returned signals. Areas with small changes in density between structures such as scar tissue and fat will produce a low reflection and be visualised as dark colours, whereas areas with significant changes in density between structures (e.g. healthy dermis) will be visualised as bright areas (Fig. 5).

An advantage of ultrasound systems are that they allow real-time measurement on changes of scar thickness upon pressure loading [112]. Additionally, high-frequency ultrasound systems will also allow the identification of aberrant structures within the scars which may affect treatment [113].

**Fig. 6:**
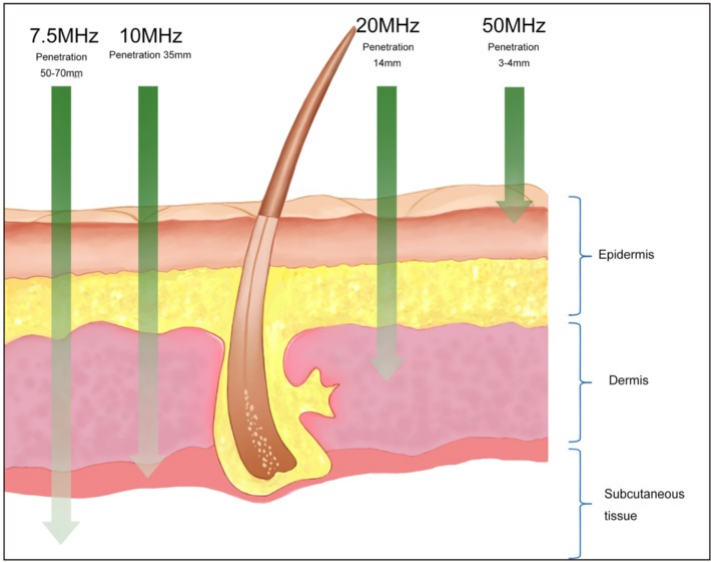
Different frequencies of ultrasounds and their penetrance into the skin. (Source: Kwang Chear Lee, adapted from image from http://www.eotech.fr/Fiches/produits/107_DUB_Brochure_English_DB10_2012_O.pdf)

The frequency of the ultrasound determines the resolution and penetrance of the measurement. A low frequency will allow deeper penetration but lower resolution images, whereas a higher frequency will have a shallower penetrance but produce higher resolution images (Fig. 6). High-frequency ultrasound systems utilise a frequency above 18 MHz to obtain images of the skin structure with acceptable resolution. In earlier studies, 7.5-MHz probes have been used to measure and track the change in thickness of healing burn scars [101, 114]. These lower frequency systems allow evaluation of deeper tissues (penetration of >15 mm) but have a low resolution of 2–3 mm which may not be sufficient for the evaluation of superficial skin structures [115]. More recently, higher frequency ultrasound probes (20 MHz) have been used to allow more detailed images of the structures of the skin to be visualised, producing higher resolutions of at least 50 μm [115–117]. Probes with frequencies below 50 MHz are advised as systems with higher frequencies and will not be able to penetrate to the average depth of hypertrophic scars which is around 4–5 mm.

It is advisable to always check with the manufacturer the actual penetrance of the systems as cheaper portable ultrasound systems (e.g. Dermalab USB Ultrasound, Cortex, Hadsund, Denmark) only penetrate a maximum of 3.4 mm despite being a 20-MHz system [6].

These high-frequency ultrasound devices both show good inter-observer reliability and moderately correlate with the modified VSS [118] (modified version of the Vancouver Scar Scale by Nedelec et al.), with the Dermascan C system having the better correlation of the two (0.41–0.50 versus 0.34). It has to be noted that the VSS measures clinical scar thickness (i.e. the thickness of the scar that is above the surface of the skin), whereas the two ultrasound systems measure histological thickness (i.e. the whole thickness of the scar above and below the surface of the skin). The Dermascan system would thus be preferred, although it is more expensive than the TUPS (however at the time of writing, there was no method to purchase the TUPS from their website). Other ultrasound systems that are commercially available include the Acuson Sequoia 512 (Siemens, Germany) [119], Episcan(Longport, USA) [120, 121] and the DUB®SkinScanner (EOTech, France) [122], although at present there are no published studies that have utilised these for scar measurement.

Ultrasound systems that can capture a 3D image of a scar have now become commercially available, albeit only from one company (Cortex, Hadsund, Denmark). However, this system has not been trialled on scars, is limited to a small measurement area (22 × 22 mm) and costs significantly more compared to the 2D system (Table 4).

**Table 4:** Comparison of ultrasound devices in terms of parameter measured, reliability, correlation with clinical score and cost

Device	Company	Parameter	Intra-rater reliability	Inter-rater reliability	Correlation with clinical score	Cost	Portability	References
Dermascan C (2D)	Cortex	Thickness (2D)	ICC = 0.91–0.93	ICC = 0.90–0.91	Modified VSS and ultrasound thickness: Spearman's *r* = 0.41–0.50	£15,000–20,000	Yes	Nedelec et al. 2008 [30, 106].
Dermalab USB (2D)	Cortex	Thickness (2D)	ICC = 0.92–0.97	ICC = 0.86–0.98	No data	<£10,000	Yes	Gankande et al. 2014 [6].
Dermascan C (3D)	Cortex	Thickness (3D)	No data	No data	No data	£30,000–40,000	Yes	N/a
Tissue ultrasound palpation system	Biomedical Ultrasonic Solutions	Thickness (2D)	ICC = 0.98	ICC = 0.84	Spearman Correlation of 0.42 between VSS thickness score and TUPS measurement (*p* < 0.01), and *r* = 0.34 (*p* < 0.01) between VSS total score and TUPS.	Not currently commercially available.	Yes	Lau et al. 2005 [103].

*2D* = two-dimensional; *3D* = three-dimensional; *ICC* = intra-class correlation coefficient; *VSS* = Vancouver Scar Scale; *TUPS* = Tissue Ultrasound Palpation System

A summary of the different ultrasound systems is given in Table 4.

### Texture

#### Skin topography

Scar roughness has a significant effect on the patient’s and observer’s opinion of the scar [4]. Indirect methods of measuring skin topography that involve creating a negative replica of the skin using materials such as polymers (e.g. Silflo silicon polymer; Flexico Developments Ltd., Hertfordshire, UK [123]) and then further analysing this with devices (e.g. mechanical, optical, laser or interference fringe projection profilometry [123–125]), although accurate can be very time consuming and not appropriate for clinical use [126]. Transparency profilometry (using the Visiometer; Courage + Khazaka, Germany) uses the Silflo silicon polymer but analysis is much easier and quicker [127, 128]. However, these indirect measurement techniques have been clinimetrically evaluated [123].

The Phaseshift Rapid In Vivo Measurement Of the Skin [129] (PRIMOS; Omniscan, GFMesstechnik GmbH; Germany) and the Visioscan VC 98 (Courage + Khazaka, Germany) are the only devices currently on the market that can be used to measure skin topography directly, but only the PRIMOS system has published studies in scars.

Three parameters were used for the evaluation of the PRIMOS system by Bloemen et al [129]. These were the peak count (PC, number of peaks per unit length), arithmetic mean of surface area roughness (Sa, in micrometers) and the mean of five highest peaks and five deepest valleys form entire measurement (Sz, in milimeters).

The PRIMOS has been shown to have excellent intra-observer and inter-observer reliability on both normal skin and scars and a high correlation with the relief score of the Patient and Observer Scar Assessment Scale (POSAS) on scar (The relief score in the POSAS questionnaire is the rating given by patients and clinicians on the surface irregularity of their scar compared to normal skin).

An added advantage of the PRIMOS system is that it can also be used to measure scar height [130].

The Visioscan VC 98 is a UVA-light video camera with high resolution that utilises the Surface Evaluation for Living Skin (SELS) method to evaluate the roughness of skin [131]. This method analyses the grey level distribution of the image captures and allows the calculation of four clinical parameters to quantitatively and qualitatively describe the skin surface as an index: skin smoothness (Sesm), skin roughness (Ser), scaliness (Sesc), wrinkles (Sew). As mentioned previously, this system has not been used to evaluate scars but has shown a high reliability for the measure of in vivo skin roughness in normal skin [131]. However, the Visioscan only measures an area of 6 × 8 mm at a time which is probably too small for the analysis of the irregularity of a burn scar.

The aforementioned 3D camera systems can potentially also be used for skin topography analysis. However, these systems are already becoming the preferred devices in the clinic for scar surface area measurement as they are significantly more portable than the PRIMOS system although portable versions of the PRIMOS system are now commercially available (PRIMOS lite, GFMesstechnik GmbH; Germany). Lumenta et al. showed that the Lifeviz Micro 3D camera system (Quantificare S.A., Sophia Antipolis, France) was able to detect surface irregularities (SI) much better than subjective visual assessment which failed to detect at least half of the broader changes in SI of ≥34 % [76]. Kim et al. utilised a self-developed 3D camera system (Stereoimage Optical Topometer, Korea University, Seoul, Korea) to calculate the mean surface area roughness (Sa) and root mean square roughness (Sq) for acne scars which were found to have a positive correlation with visual gradings (Spearman correlation coefficient *ρ* = 0.463 and 0.438 respectively, *p* < 0.001). Table 5 below summarises the surface topography devices.

**Table 5:** Comparison of surface topography measuring devices in terms of parameter measured, reliability, correlation with clinical score and cost

Device	Company	Parameter	Intra-rater reliability	Inter-rater reliability	Correlation with clinical score	Cost	Portability	References
PRIMOS	GFMesstechnik	Surface roughness (PC, Sa, Sz)	ICC of PC = 0.97, Sa = 0.99, Sz = 0.98	ICC of PC = 0.9, Sa = 0.96, Sz = 0.94	Correlation with POSAS: *r* = 0.617 (*p* < 0.001)	£17,000–£14,000	Yes	Bloemen et al. 2011 [129].
Visioscan VC 98	Courage + Khazaka	Skin parameters (Sesm, Ser, Sesc, Sew)	Not been used in scars	Not been used in scars	Not been used in scars	£5000–£10,000	Yes	N/a
Eykona 3D camera	Fuel 3D	Not been used in scars	Not been used in scars	Not been used in scars	Not been used in scars	<£5000	Yes	N/a
Lifeviz Micro	Quantificare	Surface Irregularity	No data	No data	Performed better than subjective visual assessment	£10,000–£15,000	Yes	Lumenta et al. 2011 [76].

*PRIMOS* = Phaseshift Rapid In Vivo Measurement Of the Skin; *ICC* = intra-class correlation coefficient; *PC* = peak count; *Sa* = mean surface area roughness; *Sz* = mean of five highest peaks and five deepest valleys; *POSAS* = Patient and Observer Scar Assessment Scale

### Biomechanical properties

#### Pliability, elasticity or stiffness

The biomechanical properties of skin can be measured with a variety of methods including suction, tonometry, torsion, adherence and reviscometry. Other methods include elastometry, ballistometry, quantitative electrical methods (dielectric measurements and bio-impedance) [132] as well as ultrasound and MRI techniques [133].

##### Non-suction extension methods

Older methods of measuring skin elasticity relied on extension methods (i.e. physical stretching) to measure the viscoelastic properties of skin tissue using ex vivo [134] or in vivo extensometers [135–140] or elastometers [58], which utilises a constant-tension spring and a strain gauge to distract two points on the skin [58, 141]. The majority of these devices suffer from an unwanted peripheral force contribution due to the deformation of surrounding tissues during measurement which can lead to reduced accuracy and reproducibility of results, although newer designs have sought to improve their accuracy [137].

##### Suction extension methods

**Fig. 7:**
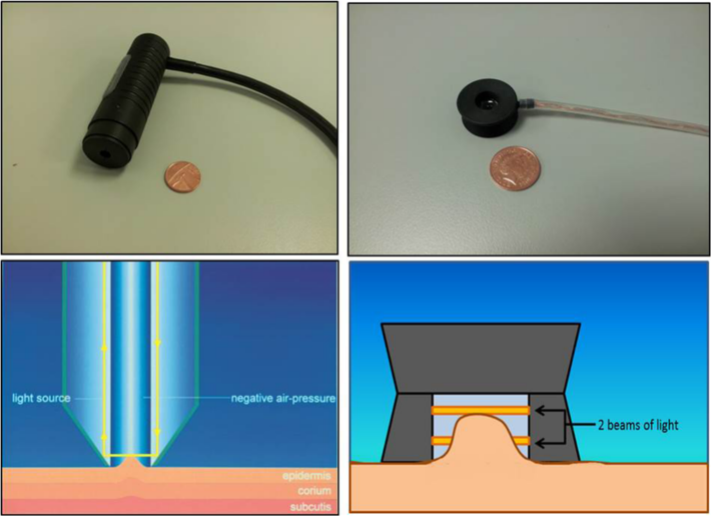
The Cutometer (*top left*) and Dermalab elasticity probe (*top right*), one penny coin to provide an idea of the size of the probes. Illustration of the mechanism of the Cutometer and Dermalab elasticity probe (*bottom left* and *right*, respectively). (Source: photographs and diagram of elasticity probe by Kwang Chear Lee; Cutometer image source: Courage + Khazaka Electronic GmbH, http://www.courage-khazaka.de/index.php/en/products/scientific/140-cutometer, reprinted with permission)

**Fig. 8:**
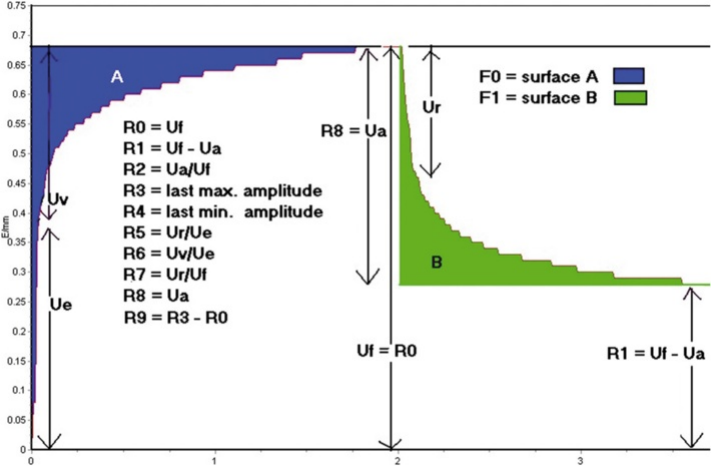
Example of skin deformation curve obtained with the Cutometer. (Source: Courage + Khazaka Electronic GmbH, reprinted with permission)

Extension of the skin by suction is the method used by devices such as the Cutometer [18, 28, 106, 142–153] (Courage + Khazaka, Germany) and the DermaLab elasticity probe [144, 154] (Cortex Technology, Hadsund, Denmark). With the Cutometer, negative pressure is created in the device by vacuum and the skin is drawn into the aperture of the probe and after a defined time is released again. Inside the probe, height of skin that is drawn up is determined by a non-contact optical measuring system which consists of a light source and a light receptor, as well as two prisms facing each other, which project the light from transmitter to receptor (Fig. 7). The resistance of the skin to the negative pressure (firmness) and its ability to return into its original position (elasticity) are displayed as curves (penetration depth in mm/time) in real time during the measurement (Fig. 8). This measurement principle allows getting information about the elastic and mechanical properties of the skin surface.

The Cutometer is reliable for measurement of the elastic and mechanical properties in scars and normal skin; however, its measurements only have a weak to moderate correlation with the pliability score of the POSAS and the subjective pliability assessment of the VSS [142]. Rennekampff et al. also suggested that the Cutometer may not be sensitive enough to pick up small changes in pliability as he found no correlation was found between subscale VSS pliability rating and Cutometer readings [155].

It was also found to be unreliable for severe scars due to a ceiling effect when rigid tissue is encountered [106]. However, the low ICC values have more to do with difficulty in relocating device to same measurement spot and the high sensitivity of the device [30, 106].

The mechanical parameters of the skin can be divided into absolute and relative parameters:

- Absolute (in milimeters): Ue (immediate deformation), Uv (delayed deformation), Uf (maximal deformation), Uf (immediate retraction), Ua (final detraction), R (residual deformation), R8 (visco part).
- Relative (in percentage): Ua/Uf (gross elasticity), Ur/Uf (biological elasticity), Ur/Ue (net elasticity), Uv/Ue (viscoelastic to elastic ratio), H (hysteresis).

Absolute parameters are likely to be influenced by skin thickness which in turn is dependent on various factors such as age, gender, anatomical region thus to compare values you will need to standardise them for skin thickness using an ultrasound and this is not always possible thus the relative parameters are more useful as it can be assumed to be independent of skin thickness which allows the values in different subjects, anatomical regions and times to be compared.

Various different opinions regarding the value that should be used (Table 6); however, Draaijers et al. concluded that either Ue or Uf is sufficient for the evaluation of scar as they found a high correlation between the parameters Ua, Ue, Uf, Ur and Uv, and that Ue and Uf were found to have the highest reliability. Nedelec et al. agreed with this and also found Uf to have a higher reliability (but not for severe scars) but concluded that as Uf is more convenient to record (automatically calculated by computer software, whereas Ue requires manual calculations), it should be used instead.

**Table 6:** Comparison of used and recommended parameters for the cutometer in different papers

Authors and papers	Parameter used/recommended
Fong et al. 1997 [146].	Uf, Ur/Uf, Ur/Ue, R8
Draaijers et al. 2004 [147].	Recommends Ue or Uf
Dobrev et al. 2005. [257]	Recommends Ue and Uf (distensibility), Ua/Uf and Ur/Uf (elasticity) and Uv and Uv/Ue (viscoelasticity)
Nedelec et al. 2008 [30, 106].	Recommends using only Uf
Rennekampff et al. 2002 [155] and 2006 [142].	Uf, Ua, Ur, Ue, Ur/Ue and Ur/Uf

Other studies have also utilised the R (dimensionless parameters derived from the U values) and Q (maximum recovery, elastic recovery and viscous recovery areas) values [143].

The Dermalab elasticity probe [6, 156] consists of a light plastic probe that is much smaller than that of the Cutometer (Fig. 5). This probe is attached to the skin using double-sided adhesive rings to form a closed chamber. Within this chamber, two narrow beams of light run at different heights parallel to the skin surface and serve as elevation detectors [154] (Fig. 5). A computer controlled vacuum pump connected to the probe is then used to increase the suction within this closed chamber over 30–60 s. In contrast to the Cutometer where a set pressure is applied and the skin deformation is measured, the Dermalab elasticity probe measures the amount of suction (in kilopascals, kPa) that is required to lift the skin to pass the height of the two light beams. This may cause problems when the measured skin is too stiff to be stretched enough to reach the level of the detectors [154]. The stiffness of the skin (or Young’s modulus, E) is then calculated and expressed in millimeter per kilopascal. Skin that is firm, e.g. scar tissue will have a higher stiffness index compared to normal skin.

A study by Gankande et al. with the Dermalab elasticity probe showed that the test–retest reliability for pliability was “excellent” (ICC 0.76–0.91) in scar areas but only “good” (ICC 0.45, 95 % CI 0.30–0.76) in contralateral normal skin areas [6]. It should be noted that significant difficulties were encountered by the researchers in the study in obtaining elasticity measurements and they failed to obtain matched measurements for test–retest analysis in 31–52 % of the subjects [6].

Both devices have the advantage of being a “hub” to which other measuring devices can be attached. For example, the Dermalab combo device provides additional probes that can be fitted to provide spectrophotometry data (melanin and erythema) and ultrasound measurement of dermal thickness [6].

#### Tonometry

Tonometry measures the firmness and flexibility of skin and scars by exerting pressure either via an airflow system that is blocked at a certain pressure (e.g. Pneumatometer [157] (Medtronic Solan Model 30 Classic, Jacksonville, FL, USA), Cicatrometer [114], Tissue Tonometer [158] (Flinders Medical Centre Biomedical Engineering, Australia) or an indentional load in a vertical direction, e.g. durometer [114, 158–161] (Rex model H 1000, Rex Gauge company, IL, USA), Schiotz tonometer [162], and Tissue Compliance Meter [163] (Model and company not stated by author). In the study by Lye et al., the Tissue Tonometer showed good intra-observer reliability and a moderate correlation with the pliability score of the VSS scale, but the measure is a relative one as it requires a contralateral reference point [158]. A study by Corica et al. [164], utilising a modified Tissue Tonometer, showed that the intra-class correlation coefficient for averaged measures between measurers (inter-rater reliability) was 0.957, and the standard error of measurement was 0.025 mm. A significant difference (*p* = .0000) between scar (2.64 ± 0.5 mm) and normal tissue (3.23 ± 0.46 mm) measurements was also demonstrated in the study. Tonometry devices are, however, less suitable for skin locations with hard bony structures underneath—as the hard underlying structures limit the degree in which the skin can be compressed. At the time of writing, the mechanical tonometer is no longer commercially available but a digital version is in the experimental phase. Other shortcomings with the mechanical design include the need to place the device accurately (must be within 5° of upright to measure correctly).

The durometer also showed good reliability and validity in one study but this was performed on sclerodermal skin [160] which demonstrates symmetrical skin thickening compared to scars where thickening can vary from area to area depending on the initial injury.

#### Torsional force and adherence measurement methods

Torsional force can be used to measure the elasticity of skin (Dermal Torque Meter; Dia-Stron, UK) [165] and the device is able to differentiate between native skin, autographs and cultured skin substitutes; however, rigorous clinical appraisals of the device have not yet been performed.

#### Acoustic methods

The Shear Velocity Device (SVD) is a portable tool that can be used to analyse soft biologic tissue by measuring the propagation of an auditory shear wave through the skin surface [166, 167]. The device works on the principal that an acoustic shear wave will have a higher velocity in a hard material (e.g. scar tissue) compared to softer material (e.g. normal skin). Experimental validation of the SVD by McHugh et al. claims that it provides similar results to the Shore Type A durometer; however, this data has yet to be published [166]. The coefficient of variation (CV) for the device in measurements of 254 hypertrophic scar locations was ±4.8 % whilst on 210 normal skin sites this was ±4.4 %. Unfortunately, the authors have not been able to locate any subsequent publications on this device and it is not currently commercially available.

Revisometry [168] (Reviscometer; Dermaviduals and Courage + Khazaka, Germany) is another portable tool that measures the elastic and viscoelastic features of skin and scars by utilising an acoustic shock wave and reports this as resonance running time (RRT). Scars have a significantly lower mean RRT compared with normal skin (52.3 versus 91.6). It has been shown to be reliable with inter-rater observer reliability of more than 0.86 on scars but more studies are required to establish its validity and comparative performance.

#### Electrical of bio-impedance methods

Utilising an impedance device, the capacitance of scar tissue has been shown to be stronger than that of normal skin and the resistance of scar tissue is lower than that of normal skin. The impedance of scar tissue however varies according to the depth and density of scar tissue [132]. This electrical property of scar tissue could be utilised to quantify scars; however, no method has been developed as of yet.

#### Modelling and other techniques

All of the methods that have been discussed thus far rely on measurements in a small area of the scar which may not be representative of the scar as a whole.

The Adheremeter [169] (Fondazione Salvatore Maugeri, Italy) uses an entirely different approach and measures the restriction of scar mobility with respect to the underlying tissue at the worst adherent point when stretched in 4 orthogonal directions using a transparent film print-out of 9 concentric rings with varying radii. It is a relatively new device and has only been tested in one study [169] but it showed an adequate level of reliability and validity when compared to the VSS. However, it has a degree of subjectivity in operation as the measurement is based on the rater’s evaluation of the force required to stretch the skin and on the patient’s judgement of comfort. It is also not suitable for use on highly concave surfaces.

A different approach to measuring the elasticity of skin is to use computerised models of skin motion analysis [170, 171]. These experimental methods are able to detect and measure the differences in elasticity between normal and scar tissue by comparing images taken at two time instances before and after deformation. Regular 2D images, combined with 3D data, can offer a method of estimating scar pliability in a more global manner [94]. In simple terms, these methods utilise a grid painted onto the skin which will then deform according to the elasticity of the skin. Grid portions that are less pliable (scar tissue) will deform less than areas which are more pliable (normal skin). A technique called Finite element modelling (FEM) can then be used to analyse this information [170–173]. This technique is still experimental and yet to be commercially available. Some devices that may be commercially available soon that utilise this technique include CutiScan CS 100 (Courage + Khazaka, Germany) which is still under development.

Other methods include the measurement of ranges of movement to determine the severity of burn contractures and thus indirectly the viscoelasticity of the scars. The current standard involves the measurement of the passive and active range of motion of an extremity in a single plane or functional movements (which are better related to activities of daily living) [174] using conventional measurements [175] (e.g. goniometry, tape measures, inclinometer) or 3D motion analysis [174, 176, 177]. The Faciometer (University of Vienna) measures the ranges of mimic movements, e.g. the distance between the tragus and the mouth using calipers and an electronic display [178]. A survey by Parry et al. however showed that there is a lack of consensus in the methods and tools used clinically for the measurement of burn contracture and these methods are also rarely checked for reliability or performance competency [175].

Table 7 gives a summary of the comparison of viscoelasticity devices in terms of parameter measured, reliability, correlation with clinical score and cost.

**Table 7:** Comparison of viscoelasticity devices in terms of parameter measured, reliability, correlation with clinical score and cost

Device	Company	Parameter	Intra-rater Reliability	Inter-rater reliability	Correlation with clinical score	Cost	Portability	References
Cutometer	Courage + Khazaka	Viscoelastic parameters	Ranges from unacceptable to good (0.12–0.76)*; poor in severe firm scars	Ranges from unacceptable to good (0.11–0.93)*, poor in severe firm scars	Low to moderate, but significant (Spearman’s *r* = −0.29 to −0.53). Rennekampf et al. could not find any significant correlation between objective viscoelastic measurements and the subjective pliability assessment of the VSS.	£5000–£10,000 (with hub)	Yes	Nedelec et al. 2008 [30, 106], Draaijers et al. 2004 [147], Rennekampf et al. 2006 [142].
Dermalab Elasticity probe	Cortex	Viscoelastic parameters	ICC = 0.90–0.93; limited ability to measure rigid scars	ICC = 0.86–0.93; limited ability to measure rigid scars	No data	£5000–£10,000 (with hub)	Yes	Gankande et al. 2014 [6].
Tonometer	Flinders Medical Centre Biomedical Engineering	Viscoelastic parameters	ICC = 0.90–0.94	ICC = 0.948	Negative correlation between VSS pliability scores and tonometer readings: −0.457 and −0.442 respectively for 3 and 6 second readings.	No longer commercially available	Yes	Corica et al. 2006 [164], Lye et al. 2006 [158].
Durometer	Rex Gauge company	Viscoelastic parameters	No data	No data for scars but ICC = 0.82–0.92 for sclerodermal skin	Good correlation with modified Rodnan skin score (0.70) for sclerodermal skin	<£1000	Yes	Merkel et al. 2008 [160].
Dermal Torque meter	Dias-tron	Viscoelastic parameters	No data	No data	No data	N/a	Yes	Boyce et al. 2000 [165].
Adheremeter	Fondazione Salvatore Maugeri	Viscoelastic parameters	Good (0.96)	Good (0.87–0.99)	Moderate correlation with VSS and Pliability subscale of VSS (rs = −0.58 to −0.66)	Free	Yes	Ferriero et al. 2010 [169].
Reviscometer	Courage + Khazaka	Resonance running time	Good (>0.86)	No data	No data	£10,000–15,000 (with hub)	Yes	Verhaegen et al. 2010 [168].
Vesmeter	Wave Cyber Co. Ltd.	No data	No data	No data	No data	N/a	Yes	Niyaz et al. 2012 [246].
Shear Velocity Device	N/a	Shear wave propagation velocity	No data for ICC. (CV for scars is ±4.8 %)	No data	No data	Not commercially available	Yes	McHugh et al. 1997 [166].

*Low ICC values for Cutometer may also be attributed to the difficulty in relocating device to same measurement spot and the high sensitivity of the device [30]

*ICC* = intra-class correlation coefficients; *CV* = coefficient of variation; *VSS* = Vancouver Scar Scale

Comparing the devices that measure biomechanical properties of scars, the Cutometer seems to be the best choice at present as it is reliable (in normal, non-hypertrophic scars), shows a reasonable validity and can be used over bony areas. Additionally, the Cutometer is the most often used device for skin viscoelasticity measurements with more publications than most of the other devices reviewed in this paper.

### Pathophysiological disturbances

Pathophysiological disturbances are defined as functional changes in the skin associated with, or resulting from, disease or injury, with measurable parameters including gas perfusion and moisture content.

#### Transcutaneous oxygen tension

Transcutaneous oxygen tension (tcpO_2_) is perturbed in injured tissues and can be used as an index of maturity in hypertrophic scars. The tcpO_2_ in scar tissue is lower compared to healthy skin, and an increase in tcpO_2_ is correlated with a reduction in scar thickness assessed both clinically and by ultrasound [179]. This is thought to be due to low oxygen diffusibility through scar tissue. A study by Ichioka et al. [180] has also shown in animal and human tissues that immature repairing tissues consumed more oxygen than mature tissues and that the oxygen consumption rate in keloid and hypertrophic scars were significantly higher when compared to mature scars which may also explain the lower tcpO_2_ in scar tissues. The method for measuring transcutaneous oxygen tension exploits the redox reactions that occur in a modified Clark electrode that measures the oxygen (tcpO_2_) and carbon dioxide (tcpCO_2_) tension on the surface of the skin. The tcpCO_2_ is considered non-specific and highly dependent of external factors, whilst the tcpO_2_ is a much more precise indicator of local perfusion [181]. This technique seems to have been recently abandoned from clinical practice.

#### Transepidermal water loss and moisture content

**Fig. 9:**
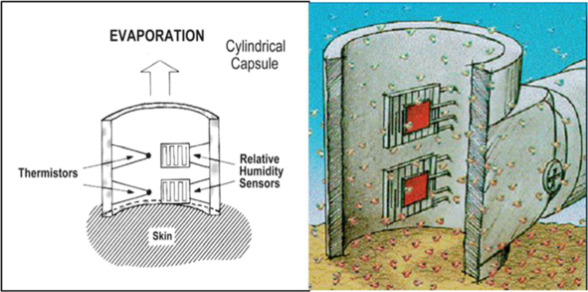
Open chamber transepidermal water loss system. (Source: Courage + Khazaka Electronic GmbH and Cortex Technology, reprinted with permission)

The water content of the skin is an important factor that influences the softness and smoothness of the skin, and transepidermal water loss and skin hydration are key indicators of skin function. Transepidermal water loss (TEWL) and moisture content can be measured by open and closed chamber systems. Open systems such as the Dermalab TEWL module [182] and Tewameter [183] (Courage + Khazaka, Germany) are the most frequently used (Fig 9). Closed systems such as the Vapometer (Delphin Technologies, Finland) are also available, but one study has shown that the Tewameter is able to detect significantly smaller differences in TEWL when compared to the Vapometer [184]. Anthonissen et al. showed a significant difference in mean TEWL values between normal skin and spontaneously healed scars (*p* = 0.036) and a significant negative relation between mean TEWL values and time after burn (*p* = 0.008); however, high SEM values were reported [156, 185].

The hydration of the skin layers, specifically the stratum corneum, can also be measured using electrical methods, such as the conductance method (for example, the Skicon-200 conductance meter [186, 187], IBS Co, Hamamatsu, Japan, Location, and the NOVA Dermal phase Meter [188], Nova, Technology Corp., Gloucester, Mass.) and impedance method (for example, the Corneometer [186], Courage + Khazaka, Germany). One study has shown that the Corneometer is suitable for use in clinical trials, with useful intra-class correlation coefficient (ICC) values (ICCintra = 0.985; ICCinter = 0.984), but only under very strict conditions with a standardised test protocol [189]. Another method for measuring hydration (and protein content) is to measure the dielectric properties of the skin. This is based on the interaction of high-frequency electromagnetic (EM) waves and biological material [190, 191]. The EM waves are generated using a network analyser (HP8753B, Agilent, USA).

A study by Suetake et al. found that TEWL was a better parameter for the functional evaluation of scars than was the hydration state of the skin surface measured by high-frequency conductometry [192].

#### Multispectral imaging systems

A novel polarised multispectral imaging system that combines out-of-plane Stokes polarimetry and Spatial Frequency Domain Imaging has been developed by Ghassemi et al. and allows the colour (haemoglobin, melanin), pathophysiology (blood oxygenation, hydration) as well as structural features (cellularity and roughness) of hypertrophic scars to be analysed in vivo [193, 194]. The results obtained with this multi-modal system showed a good agreement with the VSS and with histological examinations [193]. Although still in experimental stages, it could potentially simplify the scar measuring process due to its multi-modal measurements.

### Non-invasive morphological imaging techniques

Previously, histopathological analysis of biopsy samples was the only method of morphological investigation of damaged biological tissues. Now, recent advances in imaging techniques have made non-invasive in vivo morphological investigation of tissue microstructure possible.

#### Optical coherence tomography

With the advances in fibre optics and other technologies such as ultra-broadband light sources and frequency domain techniques, optical coherence tomography (OCT) imaging that is capable of generating 3D images of tissue microstructure is now possible. OCT is most frequently used in ophthalmology [195] but can be adapted to be used to analyse the skin [196–203]. OCT can be utilised in various different modes for the assessment of scars [202, 203]. The layered arrangement of normal skin is perturbed in scarred skin so that OCT can be used to provide information about microstructure as well as depth and volume [196]. Scar tissue imaged by OCT appears dense and bright due to the increased collagen content, and this parameter can be used to measure the collagen status of scars [204]. Scar microvasculature density has been quantified using an automated OCT system and found to be increased in hypertrophic scar tissues (38 %) when compared against normal, unscarred skin (22 %) [201]. Vessels in scars have also been shown to be much larger compared to normal skin on OCT [205]. However, due to the strong scattering and absorption of light by skin, current OCT methods are only capable of imaging to a depth of 1 to 2 mm, whereas scar thickness is usually greater than 2 mm (as determined by ultrasound) [6, 103, 206]. Nevertheless, in areas where scar tissue is thinner (e.g. in fingers), OCT (utilising the 1300-nm wavelength region) may still be useful [196]. Another way of differentiating scar tissue from normal tissue using OCT is the use of the attenuation rate, which is defined as the rate at which the OCT signal decreases with depth in the tissue [203]. Lower attenuation coefficients are seen in scarred tissue compared with normal skin tissue [203]. This method bypasses the problem of penetration depth but yields less detailed morphological data when compared to standard OCT methods. A form of OCT (termed “Polarization-sensitive Optical Frequency Domain Imaging”) can also be used to image collagen remodelling [207].

OCT imaging has been demonstrated to be feasible for use in the clinical monitoring of scar progression automated quantification of vascularity in cutaneous burn scars [195]. OCT imaging for scarring and fibrosis is currently still in its infancy and further development in the technology is required. In a study by Eraud et al. [208], although OCT was able to detect dermal nodules (which are present in hypertrophic but not keloid scars [209]) in 100 % of the specimens, it was not helpful in identifying hyalinised collagen (which is present in keloids) and cells. The technology however has the potential for tremendous growth [204].

#### Other in vivo tomography/microscopy techniques

Imaging techniques utilising specialised optical microscopes have been used to image scar tissue.

Nonlinear spectral imaging, such as multi-photon tomography based on both two-photon excited fluorescence (TPEF) and second harmonic generation (SHG), can be utilised to demonstrate the morphological structure and spectral characteristics of collagen (with SHG) and elastin fibres (with TPEF) and thus can be used to potentially distinguish hypertrophic scar tissues from normal skin and to evaluate the effects of treatments [210–216]. Information on the orientation of collagen fibres can also be investigated and analysed from these images using fast Fourier transform methods [217, 218]. Advantages these techniques have are that several extracellular matrix components and endogenous biomolecules such as collagen, keratin, melanin and elastin can be visualised in living tissue without the need for specialised processing or staining [219, 220] and high-resolution, high-contrast three-dimensional images can be obtained [220, 221].

These techniques however have similar drawbacks to OCT. The maximum depth of two-photon imaging has been reported to reach up to 1 mm in living brains [222] and thus is comparable to OCT imaging but clinical use in the skin is typically only up to 200 μm, thereby limiting its potential utility for deep scar assessment. In addition, advances in the miniaturisation of spectral imaging apparatus need to be made before it can become of practical use in a clinical setting. The multi-photon technique also has high overall system costs, a long measurement times and the inability to quantify skin redness [223]. Other non-invasive in vivo imaging techniques which currently being developed, such as confocal laser microscopy (CLM) [224, 225], also have a limited imaging depth (~300 μm) due to tissue-related aberrations and light scattering [226].

Other similar microscopy techniques include phase-contrast microtomography with synchrotron radiation technology to detect the 3D structure of dermal tissues [221].

#### Spectroscopy techniques

Another imaging method that holds future promise is the use of optical spectroscopy methods in the UV-visible-near-infrared wavelength range, including diffuse reflectance (DR) and autofluorescence (AF) spectroscopies. DR spectroscopy is based on the scattering of photons (350–800 nm) inside biological tissues due to the differences in the refractive indices and morphology of the constituents of skin such as collagen fibres. AF spectroscopy, on the other hand, is based on the fluorescence emissions from endogenous fluorophores such as collagen and elastin when excited by light in the 350–459 nm wavelength range. A combination of both spectroscopy methods increases its accuracy [227] and has been used successfully in a rabbit hypertrophic scar model with high sensitivity and specificity [228, 229]. DR spectroscopy on its own has also been shown to be able to differentiate keloids from normal skin in terms of collagen concentration, haemoglobin oxygen saturation and scattering coefficient in an in vivo human study [217] and can potentially be used to evaluate keloid scar severity [230].

#### High-frequency ultrasound systems

High-frequency ultrasound systems (such as the Dermascan and Dermalab systems [6], Cortex, Denmark) are able to provide a much greater depth of imaging (~8 mm at 20 MHz) but the resolution is inferior to OCT, CLM and MPT [196]. Pathological scars appear as easily identified echo-poor areas that are clearly distinguishable from normal skin and with densitometry analysis with dedicated software, scars are also shown to have significantly reduced densitometric values compared with normal skin (7.6 ± 4.7 versus 31.79 ± 10.8) [231]. More detailed architectural information such as collagen arrangement and cell structure cannot currently be visualised with 2D nor 3D ultrasound techniques.

#### Intravital video-capillaroscopy

Intravital video-capillaroscopy [232] is a technique that utilises an optic contact probe microscope that is attached to a computerised video microscope (e.g. Microwatcher Model VS-901, Mitsubishi Kasei Corp, Tokyo, Japan [232]) which allows photographic images of skin capillaries to be taken. Scarred skin has a deranged capillary organisation. The pictures are then scored either subjectively and/or objectively. Subjective methods score images according to angiogenic markers [232, 233] such as enlarged or tortuous loops, architectural derangement, neoangiogenesis and quantitative changes of capillary lesions. These scoring systems can be modified to allow objective quantification [234, 235]; for example, the methods used in a study by Hern et al. allowed for both non-stereological measurements (microvessel density and vessel image width) and stereological measurements (image area fraction and microvessel length density) [235]. Intravital capillaroscopic measurement of capillary density (CD) has been shown to be reliable and reproducible with a mean coefficient of intra-observer variation of CD estimate of 5.6 % and the inter-observer correlation coefficient of 0.94 [236].

A similar technique, dermoscopy, and its use in the examination of vascular structures can be a clinically useful diagnostic tool for differentiating between keloids and hypertrophic scars [237].

The dermoscopy can be used to visualise capillaries and pigmentation in the epidermal and dermal layers of the skin. An added advantage is that since dermascopes have their own light source, it is not likely to be affected by differences in environmental lighting which has been shown by Wei et al [238].

Wei et al. [238] showed that the L* (or lightness reading) from the Dermoscope (Hong Kong Productivity Council) had a significant correlation (0.448–0.536, *p* < 0.01) with the readings from the MiniScan XE Plus spectrocolorimeter (HunterLab, Reston, VA, USA) and VSS scores of pigmentation (when the skin was blanched with pressure; *r* = 0.783, *p* < 0.01). The RGB values of redness also showed a strong correlation with the VSS scores of vascularity (*r* = 0.625, *p* < 0.01). Both the intra-rater and inter-rater reliability of the dermoscope were found to be excellent (0.965–0.98 and 0.871 to 0.930, respectively).

## Measurement of sensory change

A majority of patients with burn scars experience a change in sensation of the scarred skin such as pruritis, pain and hyper- or hypo-sensitivity is common in scars and this can often last for years after the initial injury [239]. However, the objective measurement of such sensory deficits is challenging task and the only gold standard for pain assessment available currently is self-report.

Functional MRI (fMRI) scans have shown promise in assessing pain in the absence of self-report however it is far from ready for regular clinical use [240]. However, skin sensitivity/touch and (indirectly) pain can be examined in an objective manner with the touch pressure threshold method (TPT) using for example Semmes Weinstein monofilaments, which have been shown to have good intra- and inter-rater reliability (ICC = 0.822 and 0.908, respectively) in patients with scars [241].

More recently, an electronic version of the von Frey filaments is also available and showed better reproducibility compared to the traditional von Frey with good to almost perfect intra-observer reliability (ICC ranges from 0.61 to >0.8) (study done on normal skin, not scars) [242, 243].

## Discussion

There have been significant advances in many aspects of burn treatment, but hypertrophic scarring remains as one of the major chronic problems after severe burns with few therapeutic options currently available. The accurate assessment of scarring is an important aspect of research into better treatments for this condition. Despite this, scar assessment is still mostly subjective and there is still little consensus regarding the ideal scar measurement tool [244].

Most if not all currently used subjective scales used in evaluating skin scars assume that scar dimensions conform to linear models and thus employ equal appearing interval (EAI) scales. However, a study by Brandt et al. showed that whilst pliability, thickness and surface area were defined well using linear models, the dimensions of vascularity and pigmentation were more accurately described using curvilinear functions [245].

Tools for scar measurement are often modified from tools developed for other industries, e.g. dermatological use in the cosmetic industry, such as the Cutometer; colour probes for measuring the colours of materials in the food and building industries and durometers such as the Vesmeter for testing the hardness of materials in the manufacturing industry [246]. As such, their utility for burns patients is mostly unproven. Accordingly, trials on these tools to evaluate their accuracy and reliability are scarce and few trials have compared the different devices.

The ideal assessment of scars should include the objective and subjective aspects of scars as well as an assessment of the functional limitations that are caused by the scar tissues [94]. The different physical aspects of scars can all change independently of each other during the course of scar evolution and as such a hybrid method of scar assessment which incorporates the most reliable and feasible methods should be used [247]. Combination systems such as the Dermalab combo which incorporate multiple scar measurement tools (e.g. colour, thickness and pliability [6, 248]) are now available [248] to facilitate this although improvement in the clinical interpretation of the measurements is required [247].

A problem with validating objective scar measurement tools is the lack of an ideal gold standard.

Biopsies and standard histological analysis whilst proven to be accurate mostly rely on subjective scoring systems [249] unless quantitative measurement techniques are used [43]. Furthermore, Singer et al. showed that histomorphologic scales have been shown to only correlate fairly with gross macroscopic scores [249]. Beausang et al. also found that the clinical scar appearance correlated better with the upper portion of the skin (epidermis and papillary dermis) compared to the deeper parts of the scar [43]. Therefore, the lack of correlation of objective measurement techniques with clinical subjective scores should be considered carefully and not used to dismiss the objective methods.

Future validation studies of pigmentation and vascularity may be possible with standard colour reference cards developed for the cosmetic industry [250].

Objective scar measurement tools are important, especially for interventional clinical studies, as scars and the effect of therapies can be described, analysed and compared more accurately than is possible with subjective scar scales. Subjective scar scales however should still be incorporated into studies as they can provide a more global assessment of the scar and allow the measurement of variables that are currently not possible with objective measurement devices, such as pain and itch. Indeed, several published studies have incorporated both subjective and objective scar measuring tools [251, 252].

The implementation of objective measurement devices into standard clinical practice still faces many obstacles and there are multiple reasons why potentially great technologies are struggling to get incorporated into the health care system.

As mentioned previously, many new technologies (including all if not most of the devices mentioned in this review) have been developed for non-medical uses and very little if any input has been sought from clinicians or patients during the design process, and thus these devices may have limited practical clinical use. The lack of data security features is also a factor although this issue probably applies more to mobile apps rather than physical devices.

Some clinicians also view the use of new technologies in clinical practice as a crutch to the development of clinical acumen even though many studies have shown that clinical judgement to perform poorer, e.g. in determining surface area [253] or burn depth [254].

Another main issue is the high cost of these devices. Even the simplest of devices, e.g. colour probe, costs at least >£3000 not including annual servicing costs. Without solid research evidence of clinical and patient benefit, it is difficult to justify the costs and use of these devices outside of research.

Despite many of these devices being fairly simple to use, a certain level of technical expertise and additional clinical time to collect and analyse the data generated is still required. To take an example, electronic health records have been used more frequently in hospitals nowadays but it takes longer for an average clinician to input data into the electronic system than onto a paper record for months, even years, after they have started using them [255]. This has been anecdotally quoted as one of the main reasons why some burn clinicians have been slow to adopt new technologies such as LDI in determining burn depth even though there is strong evidence for its accuracy compared to clinical judgement. Staff specially trained in the use of these devices and who are responsible for training of other staff and championing their use in regular clinical practice may be the way forward [255].

Lastly, the scope of this review largely did not include journals or articles in physical sciences or engineering which may have unearthed more potentially useful objective scar measurement devices.

## Conclusions

In this review, we aimed to recommend a panel of objective scar measurement tools for burn scars to be used in conjunction with subjective scar scales, that were reliable, patient friendly, and easy to use (feasibility in terms of cost and portability have now been commented on in the tables in the various sections); generated simple data; and were appropriate for use in a clinical (bedside) environment (i.e. portable). We included in the panel the least number of devices that could measure surface area, colour, thickness, pliability, texture or topography and pathophysiological skin disturbances in order to reduce measurement time and cost. All of the devices considered for inclusion have to be commercially available. As such, the recommended device panel for burn scar assessment is as follows:

- *3D wound measurement camera systems (Eykona/Lifeviz/Vectra H1)*: for surface area, texture, volume (including clinical thickness) and colour.
- *Dermascan*: for histological thickness measurements (the TUPS is an alternative but not commercially available).
- *DSM II Colormeter*: for colour measurements (both Tristimulus reflectance colorimetry and narrow-band simple spectrophotometry).
- *Cutometer system*: for viscoelastic measurements of the skin.
- *Tewameter (optional probe for the Cutometer system)*: for the measurement of transepidermal water loss.

Further studies are needed to validate the performance and utility of this scar panel and to compare them with the commonly used subjective scar scales, such as the POSAS.

It is recommended that new technologies to be utilised in objective measurement should ideally be evaluated in terms of intra- and inter-rater reliability (with at least two observers) before being used in trials; however, this could be time and resource consuming. Collaborations should be established between the industry, clinical research and patient groups to streamline and refine this process and encourage the testing and introduction of improved devices.

Although there is a greater emphasis now compared to previous decades on developing and evaluating devices that measure physical scar parameters, scarce attention has been given to measure the physiological characteristics of scars. It is essential to develop tools that can be used to measure and quantify metabolic and cellular activity in scars so that treatments can be tailored to the individual.
